# Multiresidue analysis of basic, neutral, and acidic pesticides in biobeds’ biomixture

**DOI:** 10.1016/j.mex.2022.101697

**Published:** 2022-04-14

**Authors:** Sofía Rezende, Natalia Besil, Lucas Archondo, Horacio Heinzen, María Verónica Cesio

**Affiliations:** a Grupo de Análisis de Compuestos Traza, Departamento de Química del Litoral, CENUR Litoral Norte, Universidad de la República (UdelaR), Paysandú, Uruguay; b Grupo de Análisis de Compuestos Traza, Farmacognosia y Productos Naturales, Departamento de Química Orgánica, Facultad de Química, Universidad de la República (UdelaR), Montevideo, Uruguay; c Grupo de Análisis de Compuestos Traza, PDU “Abordaje Holístico al Impacto del uso de Agroquímicos en Alimentos y Ambiente”, CENUR Litoral Norte, Universidad de la República UdelaR, Paysandú, Uruguay; d Graduate Program in Chemistry, Facultad de Química, Universidad de la República, Montevideo, Uruguay

**Keywords:** Acidic herbicides, Biobeds, Soybeans, LC-MS/MS

## Abstract

Contain between 1 and 3 bullet points highlighting the customization rather than the steps of the procedure.

An analytical methodology was adjusted and validated for the analysis of 15 selected pesticides currently employed in extensive agriculture. The main application of this methodology is studying the pesticides degradation behavior using biobeds as a friendly environmental tool for the treatment of wastewaters generated in fields. The scope of the method was selected based on the most used pesticides in soybean crops in Uruguay. The novelty of this work is the inclusion of neutral and acidic herbicides such as 2,4-D, clethodim, dicamba, together with fungicides and insecticides which are usually included in Multi Residue Methods. An acetonitrile extraction methodology without a *clean-up* step yielded acceptable results for all the analytes. The instrumental analysis was performed using HPLC-MS/MS. The selected methodology was validated according to the SANTE guidelines. The recoveries were between 65 and 130% with RSD < 20%. The instrumental LOQs were fixed at 1 µg/L for all the compounds except for clethodim, and the method LOQs were 1 mg/kg in biomixture dry basis. These LOQs values are acceptable for biodegradation studies in biobeds.

A multiresidue methodology was:

• Validated for 15 pesticides in biomixture.

• Acidic herbicides were included in the scope.

• The method was employed for the environmental monitoring of pesticide degradation in biobeds.

## Graphical abstract

**Figure:**
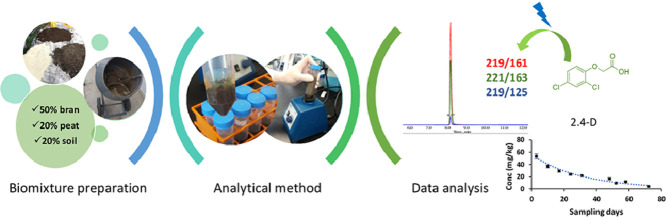

Specifications table

**Table:** 

Subject Area;	Chemistry
More specific subject area;	*Analytical chemistry, pesticide residue analysis*
Method name;	*Wide Scope Pesticide Residues (WISPER) method for BIOBEDS*
Name and reference of original method;	*QuEChERS*[1]
Resource availability;	*N.A.;*

## Sample preparation and pre-treatment

The biomixture employed as a blank was prepared by mixing topsoil, peat, and rice bran at a volumetric ratio of 1:1:2 using a concrete mixer according to previous results of our group [2]. The soil was collected in 2019 from the top 20 cm of a field site used for agriculture experiment at the agronomy experimental station EEMAC (32°22′51.46"S - 58° 3′13.72"O) belonging to a *Vertic Argiudolls* soil type, characterized by a base saturation (BS) > 60% and a cation exchange capacity (CEC) > 25 meq/100 g [3]. The soil was collected in polyethylene bags and transferred to the laboratory where they were sifted with 5 mm diameter sifter. Rice bran was acquired at a local market (CALSAL, Salto, Uruguay) and the peat was from Kekkilä Ratatie 11, Vantaa, Finland. A 10g representative sample from the prepared biomixture was lyophilized (48 h) with a freeze dryer BW-10 from Bluewave Industry Co. Ltd. (Shanghai, China), prior to be used to build the biobed to evaluate the possible presence of the studied pesticides.

## Pesticides’ selection

The analytes selected for the study were 15 pesticides normally used in Uruguay during the soybean production. Those analytes, listed on Table 1, belong to different chemical families, and have different physicochemical properties. It is noteworthy that acidic herbicides are included in the scope. The development of a specific analytical methodology for the multiresidue analysis of the targeted compounds is the first step to study their biodegradation using biobeds.

**Table 1:** Pesticides’ physicochemical properties.

Pesticide	Chemical family	Chemical structure	pKow	pka
2.4-D	Phenoxyacetic acid		2.81	2.73
Abamectin	Avermectin		4.4	-
Bifenthrin	Pyrethroid		6.0	-
Chlorantraniliprole	Carboxamide		2.76	10.88
Chlorpyrifos	Chlorinated organophophate		4.96	-
Clethodim	Cyclohexanedione		4.21	-
Dicamba	Benzoic acids		2.21	1.97
Epoxiconazole	Azol		3.44	-
Lambda cyhalothrin	Pyrethroid		5.5	-
Metolachlor	Chloroacetamide		3.4	-
Pyraclostrobin	Strobilurin		3.99	-
Saflufenacil	Pyrimidinedione		2.6	4.41
Sulfentrazone	Triazol		0.991	6.56
Thiametoxam	Neonicotinoid		-0.13	-
Triflumuron	Benzoylphenylurea		4.9	-

Analytes under study, chemical families, chemical structure, pkow and pka.

## Evaluated methods

Acetonitrile extraction of pesticides from the biomixture (Method A)

1) Weigh 2 g of freeze dried biomixture into a 50 mL polypropylene centrifuge tube and add 8 mL of H_2_O and agitate by hand for 1 min.
2) Add 10 mL of acetonitrile and agitate manually for 1 min.
3) Add 4 g of MgSO_4_ and 1 g NaCl. Shake manually vigorously during 3 min and centrifuge 5 min at 226 g.
4) Transfer a 7 mL aliquot to a 15 mL polypropylene tube containing 1050 mg MgSO_4_ (150 mg/mL). Homogenize with vortex for 30 s and centrifuge 5 min at 462g.
5) Filter 1 mL extract trough 0.22 µm PTFE filter into a 2 mL vial for LC-MS/MS analysis.

Acetonitrile extraction with higher ionic strength in acid media (Method A1)

1) Weigh 2 g of freeze dried biomixture into a 50 mL polypropylene tube and add 8 mL of H_2_O adjusted at pH = 3 with acetic acid and agitate manually for 1 min.
2) Add 10 mL of acetonitrile and agitate manually for 1 min.
3) Add 4 g of MgSO_4_ + 1 g NaCl, agitate manually vigorously for 3 min and centrifuge 5 min at 226 g.
4) Transfer an aliquot to a 15 mL polypropylene tube containing 150 mg MgSO_4_/mL. Homogenize with vortex for 30 s and centrifuge 5 min at 462 g.
5) Filter 1 mL extract trough 0.22 µm PTFE filter into a 2 mL vial for LC-MS/MS analysis.

Acetonitrile extraction with higher ionic strength (Method A2)

1) Weigh 2 g of freeze dried biomixture into a 50 mL polypropylene tube and add 8 mL of H_2_O and agitate manually for 1 min.
2) Add 10 mL of acetonitrile and agitate manually for 1 min.
3) Add 4 g of MgSO_4_ and 3 g NaCl, agitate manually vigorously for 3 min and centrifuge 5 min at 226 g.
4) Transfer a 7 mL aliquot to a 15 mL polypropylene tube containing 1050 mg MgSO_4_. Homogenize with vortex for 30 s and centrifuge 5 min at 462 g.
5) Filter 1 mL extract trough 0.22 µm PTFE filter into a 2 mL vial for LC-MS/MS analysis.

## Validation parameters

The evaluated parameters for the analytical method validation following the guidelines of the DG SANTE 2017 were trueness, precision (RSDr), limits of quantification (LOQs), linearity, and matrix effect (ME) [4,5]. For the recovery studies, 3 levels of concentration were selected: 1, 10 and 50 mg/kg. Recoveries percentages obtained for the 15 pesticides under study (*n* = 5) were averaged to estimate the trueness and the RSDr of the method; the criteria guidelines for recoveries values are in a range of 70–120% and RSDr less than 20%. These criteria were used to establish the values of LOQs as the minimum value which had a recovery between 70 and 120% and RSD < 20%. Linearity was evaluated with the back calculated concentration (BCC) obtained with the calibration curves; the acceptance criteria is BCC < ± 20%. Matrix Effect in percentage (%ME) was calculated comparing the curve in solvent with that in matrix. Matrix matched external calibration curves were used to quantify the concentration of all the studied compounds.

(1) $$\%\text{ME}=\frac{\text{Matrix}\;\text{matched}\;\text{calibration}\;\text{curve}\;\text{slope}\;-\text{solvent}\;\text{calibration}\;\text{curve}\;\text{slope}}{\text{solvent}\;\text{calibration}\;\text{cuve}\;\text{slope}}\;x100$$

## LC-MS/MS analysis

A HPLC Agilent 1200 (Agilent Technologies, Palo Alto, CA, USA) coupled to a mass spectrometer 4000 QTRAP (AB SCIEX) was employed for detection and quantification of target compounds. A ZORBAX Eclipse XDB-C18 (150 × 4.6 mm, 5 µm) column (Agilent Technologies) was used for the compound's separation. The temperature of the column oven was set at 20 °C. Two elution programs were employed. The elution program in positive ESI mode was settled as follows: 10% B was held for 3 min and increase to 100% over 20 min (5 min hold); finally, the %B came back to initial value over 3 min and it was hold for 7 min (33 min total run), using as mobile phase: water with 5 mM of ammonium formate, 20% of methanol and 0.1% of formic acid (A), and methanol with 5 mM of ammonium formate, 20% of water and 0.1% of formic acid (B).In negative acquisition mode water with 0.1% of formic acid (A) and acetonitrile (B) were used as mobile phase. The elution program in this case starts 30% B that was hold for 1 min and increase to 100% over 6 min (4 min hold) and then return to the initial conditions over 1 min and it was hold for 5 min (16 min total run). In both cases, five microliters were the injection volume, and the flow rate was set at 0.6 mL/min.

The chromatograph was coupled to a quadrupole-linear ion trap, operated in triple quadrupole MS/MS mode with an ESI source operating with positive and negative ionization modes. The ionization settings used in the positive and negative mode were ion spray voltage, 4500 V; nitrogen as curtain gas, 20 (arbitrary units); GS1, 50 psi; GS2, 50 psi; and temperature, 450 °C. In both cases, nitrogen was used as the nebulizer gas and collision gas. Multiple reaction monitoring (MRM) was settled as acquisition mode.

The MS parameters of the target analytes were optimized in the LC-MS/MS system using direct injection analysis with the individual standards. The flow rate was 10 µL/min. Table 2 summarizes compound dependent parameters. Analyst software v 1.8.1 (SCIEX) was used for data acquisition and processing.

**Table 2:** Instrumental conditions for target pesticides using LC-MS/MS.

Analytes	Q1	Q3	tr (min)	DP (V)	EP (V)	CE (V)	CXP (V)
2.4-D	218.9	124.9	8.0	-50	-10	-38	-50
		160.9				-18	-9
	220.9	162.9				-18	-9
Abamectin	890.6	305.4	25.1	75	10	34	14
		567.3				18	14
Bifenthrin	440.1	181.2	25.7	36	10	21	10
		166.2				55	10
Chlorantraniliprole	484.1	286.0	20.2	44	10	21	13
		453.0				22	24
Chlorpyrifos	350.0	198.1	24.1	80	8	23	10
		125.3		49	10	29	19
Clethodim 1	360.1	268.1	21.07	46	10	17	18
		164.1				29	10
Clethodim 2	360.1	268.1	23.21	46	10	17	18
		164.1				29	10
Dicamba	219.0	174.8	7.5	-48	-10	-9	-10
		144.8				-15	-10
	221.0	176.8		-47		-9	-15
Epoxiconazole	330.1	101.2	21.9	36	10	63	10
		121.3				27	10
Lambda cyhalothrin	467.1	225	23.9	16	10	23	12
		141.2				57	15
Metolachlor	284.1	176.1	22.1	46	10	35	10
		252.0		51		21	16
Pyraclostrobin	388.1	163.1	22.7	67	10	39	10
		194.2				17	10
Saflufenacil	501.1	459.4	20.1	82	4	20	35
		349.4				40	16
		198.1				61	33
Sulfentrazone	387.1	307	18.5	52	11	30	15
	404.2			40	6	39	13
Thiametoxam	292.0	181.2	12.8	88	10	29	10
		211.1				10	15
		246.1				10	13
Triflumuron	357.0	154.0	9.5	-16	-10	-14	-15
		175.9				-22	-15

**Fig. 1:**
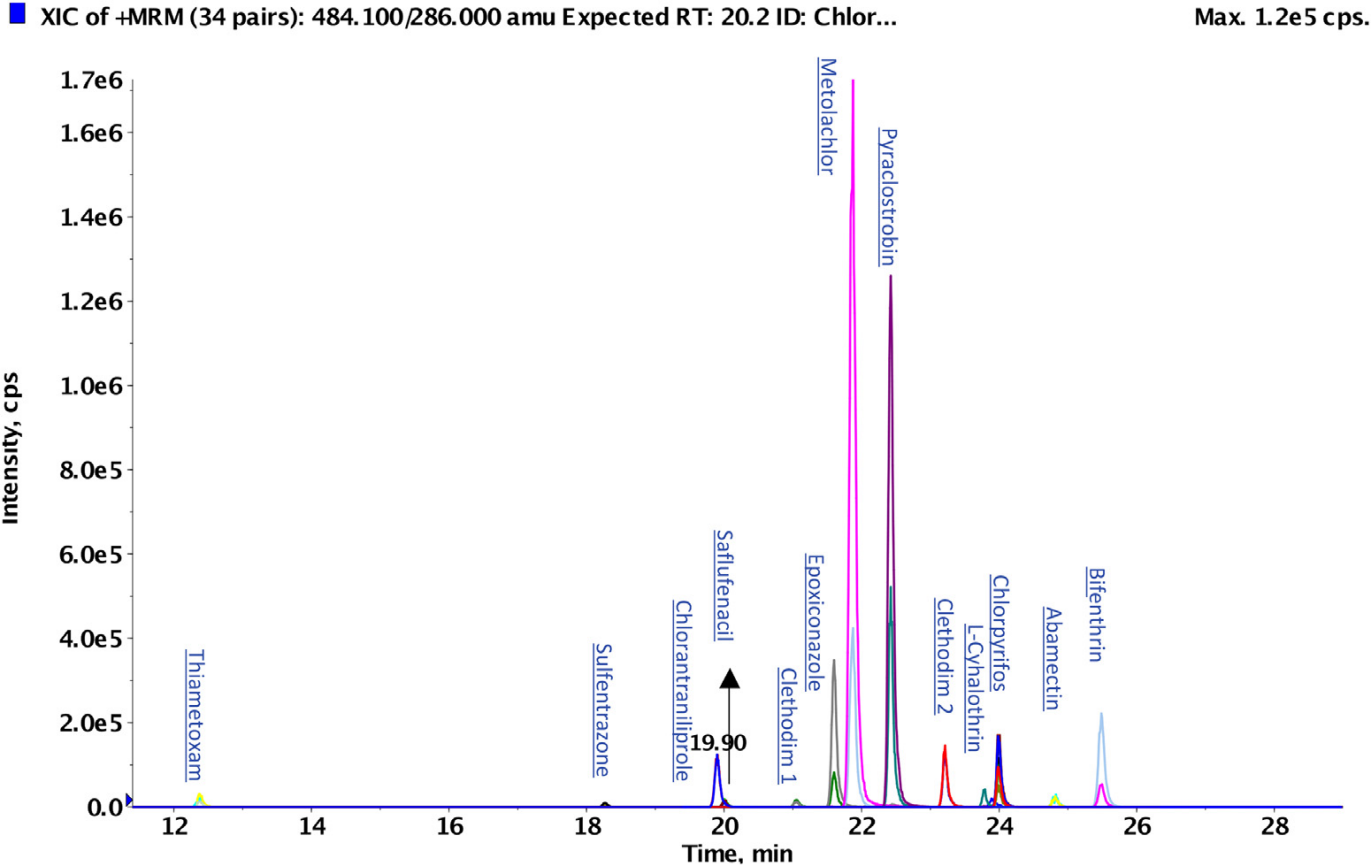
Spiked sample at 50 mg/kg analyzed by LC-MS/MS with positive acquisition mode.

Moreover, a chromatogram of a spiked sample using the described conditions above, in positive mode, is shown in Fig. 1.

## Methods comparison

Firstly, the direct acetonitrile extraction (Method A) and the two variations (A1 and A2) were compared in terms of recovery and RSD percentage at a concentration level of 1 mg/kg for the 3 pesticides that are analyzed in ESI negative mode: triflumuron, 2,4-D and dicamba.

**Fig. 2:**
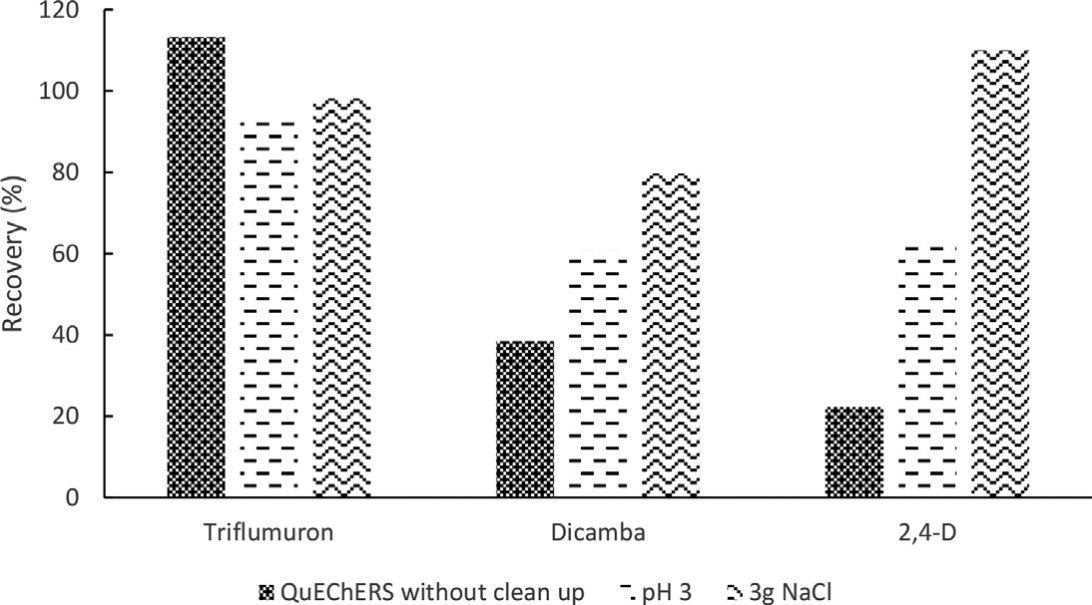
Recoveries results obtained for triflumuron, dicamba and 2,4-D with the methodologies assayed.

As it is shown in Fig. 2. The Method A showed low recoveries for 2,4-D and dicamba, 22 and 38% respectively, but acceptable ones for triflumuron (113%). Due to the acidic characteristic of these pesticides, two modifications were assayed to improve recoveries. The first modification included the addition of water at pH 3, increasing the ionic strength in the extraction media (A1). Method A1, yielded 68% for dicamba and 79% for 2,4-D recoveries respectively, but the % RSD in 2,4D determination was 30%, and triflumuron was not well detected. In the case of method A2, the amount of NaCl in the salting out step was increased to improve phase separation and no acidified water was added. Acceptable recoveries and % RSD were obtained for these for the three key compounds (Table 2). The method A2 was selected for testing the 15 pesticides for the study determined through ESI positive mode. Acceptable recoveries (75–99%) for all the pesticides with % RSD < 20% were obtained.

With these results the performance of method A2 was studied for the remaining analytes through ESI negative mode, obtaining acceptable recoveries for all pesticides. Therefore, this method was selected for the validation study.

## Validation of the analytical methodology

The validation results obtained for the evaluated parameters in this study are presented in Table 3.

**Table 3:** Validation parameters for selected pesticides using LC-MS/MS.

	Concentration levels (mg/kg)									
	1		10		50					
Pesticides	%Rec	%RSD	%Rec	%RSD	%Rec	%RSD	%ME^a^	LOQ (mg/kg)	Instrumental linear range (µg/kg)	Instrumental LOQ (µg/kg)
2.4-D	83.1	5.7	81.9	9.5	79.7	5.5	-5.7	1	1–120	1
Abamectin	118.9	6.4	110.0	13.0	90.4	6.1	-41.7	1	1–100	1
Bifenthrin	98.2	4.3	97.2	9.9	92.8	5.6	-36.2	1	1–100	1
Clethodim 1	112.3	2.4	139.4	13.1	129.6	3.5	-58.5	1	10–100	10
Clethodim 2	122.1	3.6	136.3	12.3	133.8	6.6	-14.6	1	10–100	10
Chlorantraniliprole	105.3	5.2	114.6	12.7	116.0	5.2	39.6	1	1–120	1
Chlorpyrifos	103.1	3.5	106.7	12.2	105.6	7.0	-40.5	1	1–100	1
Dicamba	68.8	10.0	64.9	11.5	64.1	6.0	7.2	1	1–120	1
Epoxiconazole	105.2	5.4	120.7	11.5	113.5	5.2	18.0	1	1–120	1
Lambda cyhalothrin	98.2	4.8	97.7	9.3	96.1	4.6	-67.4	1	1–120	1
Metolachlor	109.8	4.5	114.5	11.4	113.2	3.4	-3.1	1	1–120	1
Pyraclostrobin	108.8	4.2	114.8	11.4	112.5	5.2	-11.2	1	1–120	1
Saflufenacil	102.5	5.4	128.2	11.9	118.8	6.2	41.8	1	1–120	1
Sulfentrazone	108.6	5.9	118.5	12.8	113.7	6.6	24.2	1	1–120	1
Thiametoxam	111.0	9.0	109.7	11.7	93.9	7.7	6.6	1	1–100	1
Triflumuron	103.3	3.3	105.9	12.0	104.4	5.0	-55.6	1	1–120	1

a Matrix effect was calculated with 1/10 dilution of the extract.

**Fig. 3:**
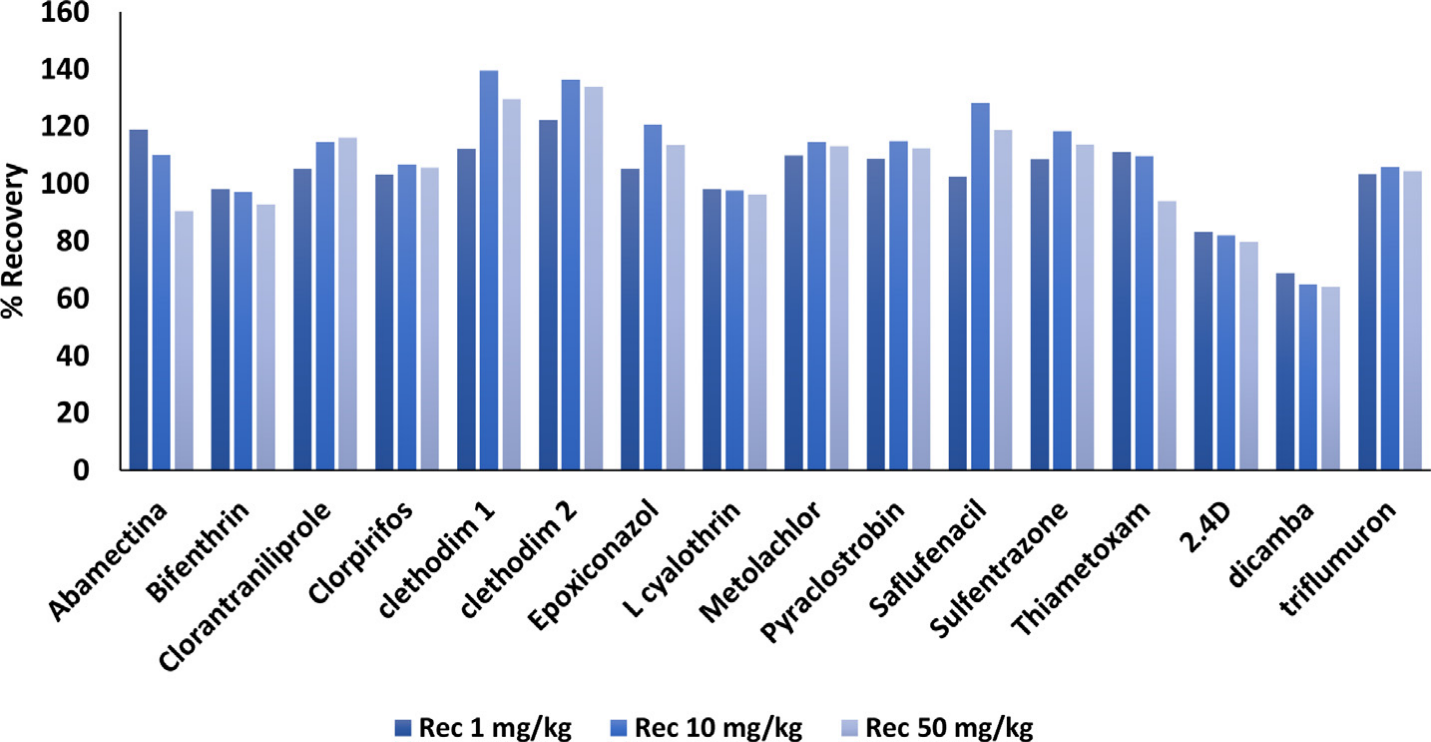
Recoveries results for the validated analytes.

As it is shown most of the pesticides presented acceptable recoveries between 79 and 120% and RSD values < 13% in all the concentration levels studied. However, dicamba showed recoveries between 64 and 69% with %RSD < 11%, this compound was included in the method because the recoveries were between 60 and 130%, and clethodim showed recoveries between 129 and 139% at the highest concentration levels studied (Fig. 3).

The LOQ reached for the method is 1 mg/kg for the analytes under study. This LOQ value is fit for purpose, due to the concentration levels obtained in biobeds after the degradation of the pesticides. Depending on the dilution factor the instrumental LOQs are settled between 1 and 10 µg/L.

Linearity was evaluated with solvent and matrix calibration curves at five concentration levels between 1 and 120 µg/L. The back calculated concentrations were calculated for all concentration levels and were lower than 20%.

Matrix effect was studied with the comparison between solvent and matrix calibration curves using the equation showed (Eq. (1)). As it was expected, the matrix effect showed suppression effects for most of the pesticides within a range between 3 and 55% (absolute value). This phenomenon could be explained due to competition during the ESI ionization step between the analytes and the matrix components [6]. However, for some pesticides such as sulfentrazone, epoxiconazole, chlorantraniliprole, thiametoxam and dicamba the matrix effect was positive but lower than 42% (Table 3).

## Declaration of Competing Interest

The authors declare that they have no known competing financial interests or personal relationships that could have appeared to influence the work reported in this paper.

## Appendix Supplementary materials

Image, application 1
