# Supplementary material for: Multiresidue analysis of basic, neutral, and acidic pesticides in biobeds’ biomixture
Source: MethodsX. 2022 Apr 14;9:101697. doi: 10.1016/j.mex.2022.101697 (PMC9061629; doi:10.1016/j.mex.2022.101697)
Supplement: Supplementary file 1 [file mmc1.docx]

**Methods article template for submitting to** [***MethodsX***](https://www.journals.elsevier.com/methodsx)

**Method Article – Title Page**

| **Title** | Multiresidue analysis of basic, neutral, and acidic pesticides in Biobeds’ biomixture. |
| --- | --- |
| **Authors** | *Sofía Rezende^a,d^, Natalia Besil^a^, Lucas Archondo^a,b^, Horacio Heinzen ^a,b^, María Verónica Cesio^a,c^(*)* |
| **Affiliations** | *^a^ Grupo de Análisis de Compuestos Traza, Departamento de Química del Litoral, CENUR Litoral Norte, Universidad de la República (UdelaR), Paysandú, Uruguay*  *^b^ Grupo de Análisis de Compuestos Traza, Farmacognosia y Productos Naturales, Departamento de Química Orgánica, Facultad de Química, Universidad de la República (UdelaR), Montevideo, Uruguay.*  *^c^Grupo de Análisis de Compuestos Traza. PDU “Abordaje holístico al impacto del uso de agroquímicos en alimentos y ambiente”, CENUR Litoral Norte. Universidad de la República UdelaR. Paysandú, Uruguay*  *^d^ Graduate Program in Chemistry, Facultad de Química, Universidad de la República, Montevideo, Uruguay.* |
| **Corresponding Author’s email address** | [*cs@fq.edu.uy*](mailto:cs@fq.edu.uy) |
| **Keywords** | - *Acidic herbicides; biobeds; soybeans; LC-MS/MS* |
| **Direct Submission or Co-Submission** | *Direct Submission* |

**ABSTRACT**

- *Max. 200 words*
- *Contain between 1 and 3 bullet points highlighting the customization rather than the steps of the procedure.*

An analytical methodology was adjusted and validated for the analysis of 15 selected pesticides currently employed in extensive agriculture. The main application of this methodology is studying the pesticides degradation behavior using biobeds as a friendly environmental tool for the treatment of wastewaters generated in fields. The scope of the method was selected based on the most used pesticides in soybean crops in Uruguay. The novelty of this work is the inclusion of neutral and acidic herbicides such as 2,4-D, clethodim, dicamba, together with fungicides and insecticides which are usually included in Multi Residue Methods. An acetonitrile extraction methodology without a *clean-up* step yielded acceptable results for all the analytes. The instrumental analysis was performed using HPLC-MS/MS. The selected methodology was validated according to the SANTE guidelines. The recoveries were between 65-130% with rsd<20%. The instrumental LOQs were fixed at 1 μg/L for all the compounds except for clethodim, and the method LOQs were 1 mg/kg in biomixture dry basis. These LOQs values are acceptable for biodegradation studies in biobeds.

A multiresidue methodology was:

- Validated for 15 pesticides in biomixture
- Acidic herbicides were included in the scope
- The method was employed for the environmental monitoring of pesticide degradation in biobeds

**SPECIFICATIONS TABLE**

| **Subject Area** | Chemistry |
| --- | --- |
| **More specific subject area** | *Analytical chemistry, pesticide residue analysis* |
| **Method name** | *Wide Scope Pesticide Residues (WISPER) method for BIOBEDS* |
| **Name and reference of original method** | *QuEChERS* [1] |
| **Resource availability** | *N.A.;* |

***Method details**

Sample preparation and pre-treatment

The biomixture employed as a blank was prepared by mixing topsoil, peat, and rice bran at a volumetric ratio of 1:1:2 using a concrete mixer according to previous results of our group [2]. The soil was collected in 2019 from the top 20 cm of a field site used for agriculture experiment at the agronomy experimental station EEMAC (32°22'51.46"S - 58° 3'13.72"O) belonging to a *Vertic Argiudolls* soil type, characterized by a base saturation (BS) > 60% and a cation exchange capacity (CEC) > 25 meq/100 g [3]. The soil was collected in polyethylene bags and transferred to the laboratory where they were sifted with 5 mm diameter sifter. Rice bran was acquired at a local market (CALSAL, Salto, Uruguay) and the peat was from Kekkilä Ratatie 11, Vantaa, Finland. A 10g representative sample from the prepared biomixture was lyophilized (48h) with a freeze dryer BW-10 from Bluewave Industry Co. Ltd. (Shanghai, China), prior to be used to build the biobed to evaluate the possible presence of the studied pesticides.

Pesticides’ selection

The analytes selected for the study were 15 pesticides normally used in Uruguay during the soybean production. Those analytes, listed on Table 1, belong to different chemical families, and have different physicochemical properties. It is noteworthy that acidic herbicides are included in the scope. The development of a specific analytical methodology for the multiresidue analysis of the targeted compounds is the first step to study their biodegradation using biobeds.

Evaluated methods

Acetonitrile extraction of pesticides from the biomixture (Method A)

1. Weigh 2 g of freeze dried biomixture into a 50 mL polypropylene centrifuge tube and add 8 mL of H_2_O and agitate by hand for 1 minute
2. Add 10 mL of acetonitrile and agitate manually for 1 minute
3. Add 4 g of MgSO_4_ and 1 g NaCl. Shake manually vigorously during 3 min and centrifuge 5 minutes at 226*g*
4. Transfer a 7 mL aliquot to a 15 mL polypropylene tube containing 1050 mg MgSO_4_ (150 mg/mL). Homogenize with vortex for 30 seconds and centrifuge 5 minutes at 462*g*
5. Filter 1 mL extract trough 0.22 μm PTFE filter into a 2 mL vial for LC-MS/MS analysis

Acetonitrile extraction with higher ionic strength in acid media (Method A1)

1. Weigh 2 g of freeze dried biomixture into a 50 mL polypropylene tube and add 8 mL of **H_2_O adjusted at pH=3** with acetic acid and agitate manually for 1 minute.
2. Add 10 mL of acetonitrile and agitate manually for 1 minute
3. Add 4 g of MgSO_4_ + **1 g NaCl**, agitate manually vigorously for 3 minutes and centrifuge 5 minutes at 226*g*
4. Transfer an aliquot to a 15 mL polypropylene tube containing 150 mg MgSO_4_/mL. Homogenize with vortex for 30 seconds and centrifuge 5 minutes at 462*g*
5. Filter 1 mL extract trough 0.22 μm PTFE filter into a 2 mL vial for LC-MS/MS analysis

Acetonitrile extraction with higher ionic strength (Method A2)

1. Weigh 2 g of freeze dried biomixture into a 50 mL polypropylene tube and add 8 mL of H_2_O and agitate manually for 1 minute
2. Add 10 mL of acetonitrile and agitate manually for 1 minute
3. Add 4 g of MgSO_4_ and **3 g NaCl**, agitate manually vigorously for 3 minutes and centrifuge 5 minutes at 226*g*
4. Transfer a 7 mL aliquot to a 15 mL polypropylene tube containing 1050 mg MgSO_4_. Homogenize with vortex for 30 seconds and centrifuge 5 minutes at 462*g*.
5. Filter 1 mL extract trough 0.22 μm PTFE filter into a 2 mL vial for LC-MS/MS analysis

Validation parameters

The evaluated parameters for the analytical method validation following the guidelines of the DG SANTE 2017 were trueness, precision (RSDr), limits of quantification (LOQs), linearity, and matrix effect (ME) [4,5]. For the recovery studies, 3 levels of concentration were selected: 1, 10 and 50 mg/kg. Recoveries percentages obtained for the 15 pesticides under study (n=5) were averaged to estimate the trueness and the RSDr of the method; the criteria guidelines for recoveries values are in a range of 70-120% and RSDr less than 20%. These criteria were used to establish the values of LOQs as the minimum value which had a recovery between 70-120% and RSD <20%. Linearity was evaluated with the back calculated concentration (BCC) obtained with the calibration curves; the acceptance criteria is BCC < ±20%. Matrix Effect in percentage (%ME) was calculated comparing the curve in solvent with that in matrix. Matrix matched external calibration curves were used to quantify the concentration of all the studied compounds.

%ME= $\frac{Matrix matched calibration curve slope -solvent calibration curve slope}{solvent calibration cuve slope} x 100$ (Eq 1)

LC-MS/MS analysis

A HPLC Agilent 1200 (Agilent Technologies, Palo Alto, CA, USA) coupled to a mass spectrometer 4000 QTRAP (AB SCIEX) was employed for detection and quantification of target compounds. A ZORBAX Eclipse XDB-C18 (150 mm × 4.6 mm, 5 µm) column (Agilent Technologies) was used for the compound’s separation. The temperature of the column oven was set at 20 ºC. Two elution programs were employed. The elution program in positive ESI mode was settled as follows: 10% B was held for 3 minutes and increase to 100% over 20 minutes (5 min hold); finally, the %B came back to initial value over 3 min and it was hold for 7 min (33 min total run), using as mobile phase: water with 5 mM of ammonium formate, 20% of methanol and 0.1% of formic acid (A), and methanol with 5 mM of ammonium formate, 20% of water and 0.1% of formic acid (B).In negative acquisition mode water with 0.1% of formic acid (A) and acetonitrile (B) were used as mobile phase. The elution program in this case starts 30% B that was hold for 1 min and increase to 100% over 6 minutes (4 min hold) and then return to the initial conditions over 1 minute and it was hold for 5 minutes (16 min total run). In both cases, five microliters were the injection volume, and the flow rate was set at 0.6 mL/min.

The chromatograph was coupled to a quadrupole-linear ion trap, operated in triple quadrupole MS/MS mode with an ESI source operating with positive and negative ionization modes. The ionization settings used in the positive and negative mode were ion spray voltage, 4500 V; nitrogen as curtain gas, 20 (arbitrary units); GS1, 50 psi; GS2, 50 psi; and temperature, 450 °C. In both cases, nitrogen was used as the nebulizer gas and collision gas. Multiple reaction monitoring (MRM) was settled as acquisition mode.

The MS parameters of the target analytes were optimized in the LC-MS/MS system using direct injection analysis with the individual standards. The flow rate was 10 μL/min. Table 2 summarizes compound dependent parameters. Analyst software v 1.8.1 (SCIEX) was used for data acquisition and processing.

Moreover, a chromatogram of a spiked sample using the described conditions above, in positive mode, is shown in Figure 1.

Methods comparison

Firstly, the direct acetonitrile extraction (Method A) and the two variations (A1 and A2) were compared in terms of recovery and RSD percentage at a concentration level of 1 mg/kg for the 3 pesticides that are analyzed in ESI negative mode: triflumuron, 2,4-D and dicamba.

As it is shown in Figure 2. The Method A showed low recoveries for 2,4-D and dicamba, 22 and 38% respectively, but acceptable ones for triflumuron (113%). Due to the acidic characteristic of these pesticides, two modifications were assayed to improve recoveries. The first modification included the addition of water at pH 3, increasing the ionic strength in the extraction media (A1). Method A1, yielded 68% for dicamba and 79% for 2,4-D recoveries respectively, but the % RSD in 2,4D determination was 30%, and triflumuron was not well detected. In the case of method A2, the amount of NaCl in the salting out step was increased to improve phase separation and no acidified water was added. Acceptable recoveries and % RSD were obtained for these for the three key compounds (Table 2). The method A2 was selected for testing the 15 pesticides for the study determined through ESI positive mode. Acceptable recoveries (75-99%) for all the pesticides with % RSD < 20% were obtained.

With these results the performance of method A2 was studied for the remaining analytes through ESI negative mode, obtaining acceptable recoveries for all pesticides. Therefore, this method was selected for the validation study.

Validation of the analytical methodology

The validation results obtained for the evaluated parameters in this study are presented in Table 3.

As it is shown most of the pesticides presented acceptable recoveries between 79-120% and RSD values < 13% in all the concentration levels studied. However, dicamba showed recoveries between 64-69% with %RSD < 11%, this compound was included in the method because the recoveries were between 60-130%, and clethodim showed recoveries between 129-139% at the highest concentration levels studied (Figure 3).

The LOQ reached for the method is 1 mg/kg for the analytes under study. This LOQ value is fit for purpose, due to the concentration levels obtained in biobeds after the degradation of the pesticides. Depending on the dilution factor the instrumental LOQs are settled between 1-10 μg/L.

Linearity was evaluated with solvent and matrix calibration curves at five concentration levels between 1-120 μg/L. The back calculated concentrations were calculated for all concentration levels and were lower than 20%.

Matrix effect was studied with the comparison between solvent and matrix calibration curves using the equation showed (eq 1). As it was expected, the matrix effect showed suppression effects for most of the pesticides within a range between 3-55% (absolute value). This phenomenon could be explained due to competition during the ESI ionization step between the analytes and the matrix components [6]. However, for some pesticides such as sulfentrazone, epoxiconazole, chlorantraniliprole, thiametoxam and dicamba the matrix effect was positive but lower than 42% (Table 3).

**Acknowledgements:**

The Join Centre FAO/IAEA/RALACA for the purchasing of the Standards. This work was supported by the National Agency for Investigation and Innovation under Grant ANII-FMV-2017-1-136757. The authors are thankfully to PEDECIBA Química for the research support to SR, NB, HH and MVC.

**Declaration of interests:** *[MANDATORY – Delete as appropriate]*

The authors declare that they have no known competing financial interests or personal relationships that could have appeared to influence the work reported in this paper.

**Supplementary material *and/or* Additional information:**

The biobeds are an environmentally friendly tool which looks for solving punctual contamination problems due to the generated wastewater in some agricultural practices as concentrated pesticides dilutions and cleaning of the application equipment. This technology was developed by a Swedish group and consist in a biomixture which contains straw, peat and soil in 2:1:1 proportion[7].

Particularly, the  present work was developed using a novel biomixture previously assayed by our group that showed the best performance in uruguayan conditions[8]. The importance of developing an analytical method to ascertain pesticides degradation is to prove the dissipation efficiency of biobeds towards different organic contaminants, commonly used in soybean plantations. The analytical methodology must be capable of following this degradation with accuracy and precision through time. This manuscript reports for the first time a study of the degradation of herbicides, fungicides and insecticides applied together using an in-house biobed.The performance of biobeds was evaluated through the decrease of the concentration of the contaminants in the study to elaborate a degradation curve for each one. The results were supported by a validated analytical methodology fit for this purpose. This analytical methodology was adjusted and validated to follow the degradation of the selected analytes in a controlled experiment with biobeds. In this case, a concentration of 50 mg/kg was selected for the pesticide’s application, for that reason the recoveries levels were settled at 1-10-50 mg/kg.

***References:** *[Include at least one reference, to the original publication of the method you customized.]*

[1] M. Anastassiades, S.J. Lehotay, D. Štajnbaher, F.J. Schenck, Journal of AOAC International 86 (2003) 412–431.

[2] A. Rivero, N. Gérez, F. Jesús, S. Niell, M. Pía Cerdeiras, H. Heinzen, M.V. Cesio, International Journal of Environmental Analytical Chemistry (2020).

[3] D. Panario, R. Puentes, Carta de Reconocimiento de Suelos Del Uruguay. Tomo I. Clasificación de Suelos, 1976.

[4] European Commission, (2017) 1–53.

[5] M.G. Valverde, M.J. Martínez Bueno, M. del M. Gómez-Ramos, F.J. Díaz-Galiano, A.R. Fernández-Alba, MethodsX 8 (2021).

[6] N. Besil, V. Cesio, H. Heinzen, A.R. Fernandez-Alba, Journal of Agricultural and Food Chemistry 65 (2017) 4819–4829.

[7] M.D.P. Castillo, L. Torstensson, J. Stenström, Journal of Agricultural and Food Chemistry 56 (2008) 6206–6219.

[8] A. Rivero, S. Niell, M.P. Cerdeiras, H. Heinzen, M.V. Cesio, Talanta 153 (2016) 17–22.

Tables and figures

Table 1. Pesticides’ physicochemical properties

| Pesticide | Chemical family | Chemical structure | pKow | pka |
| --- | --- | --- | --- | --- |
| 2.4-D | Phenoxyacetic acid | 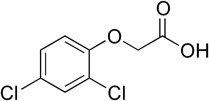 | 2.81 | 2.73 |
| Abamectin | Avermectin | 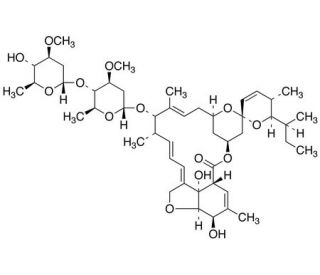 | 4.4 | - |
| Bifenthrin | Pyrethroid | 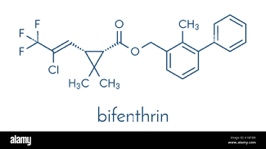 | 6.0 | - |
| Chlorantraniliprole | Carboxamide | 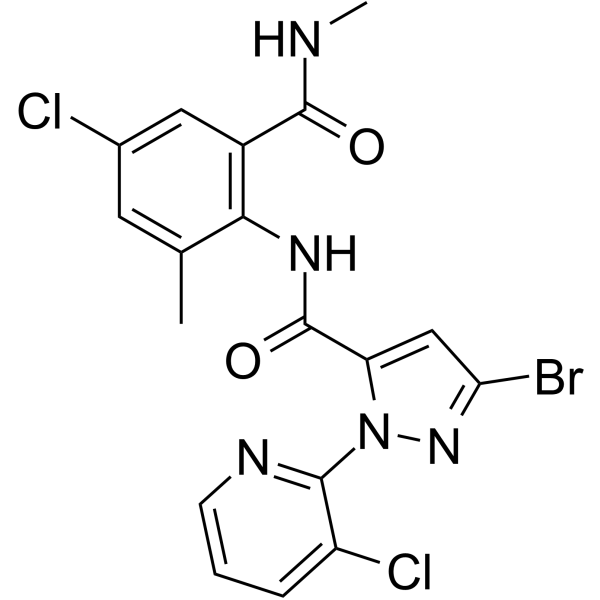 | 2.76 | 10.88 |
| Chlorpyrifos | Chlorinated organophophate | 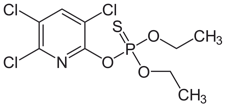 | 4.96 | - |
| Clethodim | Cyclohexanedione | 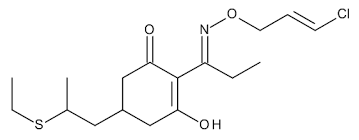 | 4.21 | - |
| Dicamba | Benzoic acids | 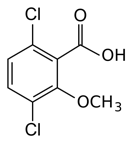 | 2.21 | 1.97 |
| Epoxiconazole | Azol | 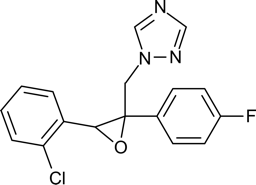 | 3.44 | - |
| Lambda cyhalothrin | Pyrethroid | 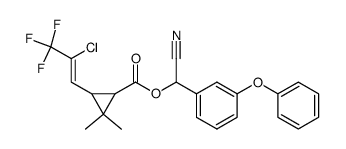 | 5.5 | - |
| Metolachlor | Chloroacetamide | 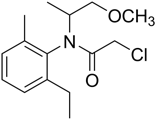 | 3.4 | - |
| Pyraclostrobin | Strobilurin | 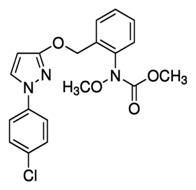 | 3.99 | - |
| Saflufenacil | Pyrimidinedione | 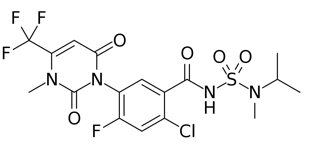 | 2.6 | 4.41 |
| Sulfentrazone | Triazol | 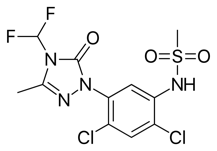 | 0.991 | 6.56 |
| Thiametoxam | Neonicotinoid | 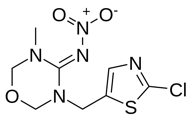 | -0.13 | - |
| Triflumuron | Benzoylphenylurea | 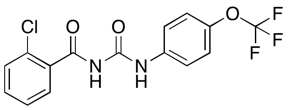 | 4.9 | - |

Table 1. Analytes under study, chemical families, chemical structure, pkow and pka

**Table 2. Instrumental conditions**

| **Analytes** | **Q1** | **Q3** | **tr (min)** | **DP (V)** | **EP (V)** | **CE (V)** | **CXP (V)** |
| --- | --- | --- | --- | --- | --- | --- | --- |
| 2.4-D | 218.9 | 124.9 | 8.0 | -50 | -10 | -38 | -50 |
|  |  | 160.9 |  |  |  | -18 | -9 |
|  | 220.9 | 162.9 |  |  |  | -18 | -9 |
| Abamectin | 890.6 | 305.4 | 25.1 | 75 | 10 | 34 | 14 |
|  |  | 567.3 |  |  |  | 18 | 14 |
| Bifenthrin | 440.1 | 181.2 | 25.7 | 36 | 10 | 21 | 10 |
|  |  | 166.2 |  |  |  | 55 | 10 |
| Chlorantraniliprole | 484.1 | 286.0 | 20.2 | 44 | 10 | 21 | 13 |
|  |  | 453.0 |  |  |  | 22 | 24 |
| Chlorpyrifos | 350.0 | 198.1 | 24.1 | 80 | 8 | 23 | 10 |
|  |  | 125.3 |  | 49 | 10 | 29 | 19 |
| Clethodim 1 | 360.1 | 268.1 | 21.07 | 46 | 10 | 17 | 18 |
|  |  | 164.1 |  |  |  | 29 | 10 |
| Clethodim 2 | 360.1 | 268.1 | 23.21 | 46 | 10 | 17 | 18 |
|  |  | 164.1 |  |  |  | 29 | 10 |
| Dicamba | 219.0 | 174.8 | 7.5 | -48 | -10 | -9 | -10 |
|  |  | 144.8 |  |  |  | -15 | -10 |
|  | 221.0 | 176.8 |  | -47 |  | -9 | -15 |
| Epoxiconazole | 330.1 | 101.2 | 21.9 | 36 | 10 | 63 | 10 |
|  |  | 121.3 |  |  |  | 27 | 10 |
| Lambda cyhalothrin | 467.1 | 225 | 23.9 | 16 | 10 | 23 | 12 |
|  |  | 141.2 |  |  |  | 57 | 15 |
| Metolachlor | 284.1 | 176.1 | 22.1 | 46 | 10 | 35 | 10 |
|  |  | 252.0 |  | 51 |  | 21 | 16 |
| Pyraclostrobin | 388.1 | 163.1 | 22.7 | 67 | 10 | 39 | 10 |
|  |  | 194.2 |  |  |  | 17 | 10 |
| Saflufenacil | 501.1 | 459.4 | 20.1 | 82 | 4 | 20 | 35 |
|  |  | 349.4 |  |  |  | 40 | 16 |
|  |  | 198.1 |  |  |  | 61 | 33 |
| Sulfentrazone | 387.1 | 307 | 18.5 | 52 | 11 | 30 | 15 |
|  | 404.2 |  |  | 40 | 6 | 39 | 13 |
| Thiametoxam | 292.0 | 181.2 | 12.8 | 88 | 10 | 29 | 10 |
|  |  | 211.1 |  |  |  | 10 | 15 |
|  |  | 246.1 |  |  |  | 10 | 13 |
| Triflumuron | 357.0 | 154.0 | 9.5 | -16 | -10 | -14 | -15 |
|  |  | 175.9 |  |  |  | -22 | -15 |

Table 2. Instrumental conditions for target pesticides using LC-MS/MS

| Table 3. Validation parameters | **Concentration levels (mg/kg)** | | | | | |  |  |  |  |
| --- | --- | --- | --- | --- | --- | --- | --- | --- | --- | --- |
|  | **1** | | **10** | | **50** | |  |  |  |  |
| **Pesticides** | **%Rec** | **%RSD** | **%Rec** | **%RSD** | **%Rec** | **%RSD** | **%ME^a^** | **LOQ (mg/kg)** | **Instrumental linear range (μg/kg)** | **Instrumental LOQ (μg/kg)** |
| 2.4-D | 83.1 | 5.7 | 81.9 | 9.5 | 79.7 | 5.5 | -5.7 | 1 | 1-120 | 1 |
| Abamectin | 118.9 | 6.4 | 110.0 | 13.0 | 90.4 | 6.1 | -41.7 | 1 | 1-100 | 1 |
| Bifenthrin | 98.2 | 4.3 | 97.2 | 9.9 | 92.8 | 5.6 | -36.2 | 1 | 1-100 | 1 |
| Clethodim 1 | 112.3 | 2.4 | 139.4 | 13.1 | 129.6 | 3.5 | -58.5 | 1 | 10-100 | 10 |
| Clethodim 2 | 122.1 | 3.6 | 136.3 | 12.3 | 133.8 | 6.6 | -14.6 | 1 | 10-100 | 10 |
| Chlorantraniliprole | 105.3 | 5.2 | 114.6 | 12.7 | 116.0 | 5.2 | 39.6 | 1 | 1-120 | 1 |
| Chlorpyrifos | 103.1 | 3.5 | 106.7 | 12.2 | 105.6 | 7.0 | -40.5 | 1 | 1-100 | 1 |
| Dicamba | 68.8 | 10.0 | 64.9 | 11.5 | 64.1 | 6.0 | 7.2 | 1 | 1-120 | 1 |
| Epoxiconazole | 105.2 | 5.4 | 120.7 | 11.5 | 113.5 | 5.2 | 18.0 | 1 | 1-120 | 1 |
| Lambda cyhalothrin | 98.2 | 4.8 | 97.7 | 9.3 | 96.1 | 4.6 | -67.4 | 1 | 1-120 | 1 |
| Metolachlor | 109.8 | 4.5 | 114.5 | 11.4 | 113.2 | 3.4 | -3.1 | 1 | 1-120 | 1 |
| Pyraclostrobin | 108.8 | 4.2 | 114.8 | 11.4 | 112.5 | 5.2 | -11.2 | 1 | 1-120 | 1 |
| Saflufenacil | 102.5 | 5.4 | 128.2 | 11.9 | 118.8 | 6.2 | 41.8 | 1 | 1-120 | 1 |
| Sulfentrazone | 108.6 | 5.9 | 118.5 | 12.8 | 113.7 | 6.6 | 24.2 | 1 | 1-120 | 1 |
| Thiametoxam | 111.0 | 9.0 | 109.7 | 11.7 | 93.9 | 7.7 | 6.6 | 1 | 1-100 | 1 |
| Triflumuron | 103.3 | 3.3 | 105.9 | 12.0 | 104.4 | 5.0 | -55.6 | 1 | 1-120 | 1 |

Table 3. Validation parameters for selected pesticides using LC-MS/MS

^a^Matrix effect was calculated with 1/10 dilution of the extract

Figure 1. Spiked sample chromatogram

Chlorantraniliprole

Sulfentrazone

Thiametoxam

Saflufenacil

Clethodim 1

Epoxiconazole

Metolachlor

Pyraclostrobin

Clethodim 2

Chlorpyrifos

Abamectin

L-Cyhalothrin

Bifenthrin

Figure 1. Spiked sample at 50 mg/kg analysed by LC-MS/MS with positive acquisition mode.

Figure 2. Comparison between the evaluated methods

Figure 2. Recoveries results obtained for triflumuron, dicamba and 2,4-D with the methodologies assayed.

Figure 3. Summary of validation recoveries

Figure 3. Recoveries results for the validated analytes
